# Profile of the Nasopharyngeal Microbiota Affecting the Clinical Course in COVID-19 Patients

**DOI:** 10.3389/fmicb.2022.871627

**Published:** 2022-05-17

**Authors:** Ornella la fortune Tchoupou Saha, Grégory Dubourg, Abdourahamane Yacouba, Vincent Bossi, Didier Raoult, Jean-Christophe Lagier

**Affiliations:** ^1^Aix-Marseille Université, Institut de Recherche pour le Développement (IRD), Assistance Publique - Hôpitaux de Marseille (AP-HM), Microbes Evolution Phylogeny and Infections (MEPHI), Marseille, France; ^2^IHU Méditerranée Infection, Marseille, France

**Keywords:** COVID-19, SARS-CoV-2, nasopharyngeal microbiota, 16S rRNA sequencing, metagenomics

## Abstract

While populations at risk for severe SARS-CoV-2 infections have been clearly identified, susceptibility to the infection and its clinical course remain unpredictable. As the nasopharyngeal microbiota may promote the acquisition of several respiratory infections and have an impact on the evolution of their outcome, we studied the nasopharyngeal microbiota of COVID-19 patients in association with baseline disease-related clinical features compared to that of patients tested negative. We retrospectively analyzed 120 nasopharyngeal pseudonymized samples, obtained for diagnosis, divided into groups (infected patients with a favorable outcome, asymptomatic, and deceased patients) and patients tested negative for SARS-CoV-2, by using Illumina-16S ribosomal ribonucleic acid (rRNA) sequencing and specific polymerase chain reaction (PCR) targeting pathogens. We first found a depletion of anaerobes among COVID-19 patients, irrespective of the clinical presentation of the infection (*p* < 0.029). We detected 9 taxa discriminating patients tested positive for SARS-CoV-2 from those that were negative including *Corynebacterium propinquum/pseudodiphtericum* (*p* ≤ 0.05), *Moraxella catarrhalis* (*p* ≤ 0.05), *Bacillus massiliamazoniensis* (*p* ≤ 0.01), *Anaerobacillus alkalidiazotrophicus* (*p* ≤ 0.05), *Staphylococcus capitis* subsp. *capitis* (*p* ≤ 0.001), and *Afipia birgiae* (*p* ≤ 0.001) with 16S rRNA sequencing, and *Streptococcus pneumoniae* (*p* ≤ 0.01), *Klebsiella pneumoniae* (*p* ≤ 0.01), and *Enterococcus faecalis* (*p* ≤ 0.05) using real-time PCR. By designing a specific real-time PCR, we also demonstrated that *C. propinquum* is decreased in asymptomatic individuals compared to other SARS-CoV 2 positive patients. These findings indicate that the nasopharyngeal microbiota as in any respiratory infection plays a role in the clinical course of the disease. Further studies are needed to elucidate the potential role in the clinical course of the disease of *M. catarrhalis, Corynebacterium accolens*, and more specifically *Corynebacterium propinquum/diphteriticum* in order to include them as predictors of the severity of COVID-19.

## Introduction

The emerging viral pathology due to the new coronavirus that appeared in Wuhan in China in December 2019 is the cause of a public health crisis that threatens humanity (Na et al., 2020; Dahyot et al., 2021). It presents a very significant clinical symptomatology; that of a clinical course characterized by pneumonia of variable severity associated with age and underlying medical conditions (cancer, diabetes, immunosuppression, obesity, cardiac, renal, respiratory, hepatic, and neurological problems) (Allameh et al., 2020; Ding et al., 2020). While populations at risk of severe SARS-CoV-2 infection have been clearly identified, the susceptibility to infection and clinical course of the disease remain unpredictable.

Many mechanisms have been associated with disease progression, from a virological, immunological, and hemostatic point of view (Gautret et al., 2020). Since the SARS-CoV-2 primary site of infection is nasopharyngeal, upon entry into the host cell it binds primarily to angiotensin converting enzyme 2 (ACE2) receptors expressed on alveolar epithelial cells of the lung and on enterocytes of the gut (Hoffmann et al., 2020; Lundstrom et al., 2021), thus communicating with the nasopharyngeal and gut microbiota (Dhar and Mohanty, 2020). At the same time, it is currently known that the nasopharyngeal microbiota may promote the acquisition of several respiratory infections that have an impact on disease progression (Dubourg et al., 2019). Research in this area has grown exponentially with the advent of high-throughput sequencing approaches (Weinstock, 2012) that allow comprehensive exploration of the nasopharyngeal microbiota in a large population of individuals (Bogaert et al., 2011; Brugger et al., 2012; Edouard et al., 2018; Man et al., 2019). Indeed, associations between changes in the nasopharyngeal microbiota and bronchiolitis, influenza virus, or human rhinovirus have been reported (Allen et al., 2014; Hasegawa et al., 2016; Wen et al., 2018). Currently, studies conducted on the nasopharyngeal microbiota of COVID-19 patients show conflicting data, and very few studies have investigated the nasopharyngeal microbiota in these COVID-19 patients, mainly using 16S ribosomal RNA sequencing (De Maio et al., 2020; Mostafa et al., 2020; Zhang et al., 2020; Lloréns-Rico et al., 2021; Rosas-Salazar et al., 2021; Shilts et al., 2021). However, no previous studies have evaluated the association between disease severity and changes in the nasopharyngeal microbiota at different disease phases.

In this context, we therefore proposed to compare the nasopharyngeal microbiota of COVID-19 patients with that of uninfected individuals and to explore the taxonomic differences among COVID-19 patients according to the disease outcome (i.e., death or survival).

## Materials and Methods

### Patients and Materials

During of COVID-19 outbreak, we tested people presenting in IHU Méditerranée Infection for a diagnosis of COVID-19 (patients with symptoms or contact of COVID-19 infected patient) and/or respiratory infection using nasopharyngeal samples. Indeed, this study was conducted on a remaining material sampled for diagnosis, and patients were not specifically sampled for this investigation.

Samples were pseudonymized and we categorized two groups: COVID-19 negative and COVID-19 positive groups, confirmed by qualitative RT-PCR assay for the qualitative detection of SARS-CoV-2 nucleic acid in upper respiratory tract samples from COVID-19 suspects (Han et al., 2021). The distribution of individuals by group was done by taking into account the basic clinical characteristics related to COVID-19 disease (fever, dry cough, dyspnea, dysgeusia, anosmia, and hyposmia) (Allameh et al., 2020). The COVID-19 group was further divided into three subgroups; asymptomatic (patients without clinical symptoms), mild (patients who had positive responses to treatment after hospitalization), and death (patients who died because of severe and critical clinical symptoms). For this study, we used the remaining amount of nasopharyngeal samples obtained following COVID-19 testing by qPCR in our institute. We performed nucleic acid extraction using the EZ1 Virus Mini Kit v2. 0 on an EZ1 Advanced XL instrument (Qiagen, Courtaboeuf, France) or the KingFisher Flex system (Thermo Fisher Scientific, Waltham, MA, United States)^®^ and for viral RNA amplification was carried out using the Master LightCycler 480 Probes kit (Roche Diagnostics, Meylan, France), as previously described (Colson et al., 2020). No additional specimen was collected as a part of this study. The study was validated by the Ethics Committee of the Méditerranée Infection Institute, under agreement number No. 2022-011. Patients were pseudonymized at the beginning of the study.

### DNA Extraction and 16S rRNA Sequencing

Samples were extracted using two methods, as previously described (Angelakis et al., 2016). Briefly, a mechanical treatment was performed with powder glass beads, acid-washed (G4649-500g Sigma-Aldrich, St. Louis, United States) and 0.5 mm glass beads cell disruption media (Scientific Industries, Inc., New York, United States) using a FastPrep-24™ 5G Grinder (MP Biomedicals LLC, Corp., Irvine, CA, United States) at maximum speed (6.5 m/sec) for 90 s. Then the samples were treated with two kinds of lysis methods: method 1, with standard lysis and protease step, followed by purification on E.Z.N.A Tissue DNA Kit (Omega Bio-Tek, Norcross, GA, United States), and method 5, using a deglycosylation step and purification on the EZ1 Advanced XL device (Qiagen, Courtaboeuf, France) (Edouard et al., 2018).

Samples were then amplified, pooled, barcoded, and sequenced using Illumina MiSeq technology (Illumina, Inc., San Diego, CA, United States) with paired-end strategy, constructed according to the 16S Metagenomic Sequencing Library Preparation (Illumina).

For each protocol extraction, metagenomic DNA was amplified for the 16S “V3–V4” regions by polymerase chain reaction (PCR) for 45 cycles, using the Kapa HiFi Hotstart ReadyMix 2x (Kapa Biosystems Inc., Wilmington, MA, United States), and the surrounding conserved region V3_V4 primers with overhang adapters FwOvAd_341F

TCGTCGGCAGCGTCAGATGTGTATAAGAGACAGCCTA CGGGNGGCWGCAG; RevOvAd_785R,

GTCTCGTGGGCTCGGAGATGTGTATAAGAGACAGGAC TACHVGGGTATCTAATCC. After purification on AMPure beads (Beckman Coulter Inc., Fullerton, CA, United States), the concentration was measured using high sensitivity Qubit technology (Beckman Coulter Inc., Fullerton, CA, United States) and dilution to 3.5 ng/μL was performed. At this step, the library of protocol 1 was pooled volume-to-volume to the library for protocol 5, and Illumina sequencing adapters and dual-index barcodes were added to the amplicon. After purification on AMPure beads (Beckman Coulter Inc., Fullerton, CA, United States), the first library was pooled with 95 multiplexed samples and the second library with 41 multiplexed samples. The global concentration was quantified by a Qubit assay with the high sensitivity kit (Life Technologies, Carlsbad, CA, United States).

Before loading for sequencing on MiSeq (Illumina Inc., San Diego, CA, United States) the pool was diluted at 8 pM. Automated cluster generation and paired-end sequencing with dual index reads were performed in a single 39-h run in a 2 bp × 250 bp.

The paired reads were filtered according to the read qualities. The raw data were configured in fastq files for R1 and R2 reads (Edouard et al., 2018).

### Identification of Respiratory Bacteria

#### Real Time PCR

Real-time PCR amplification was performed using the Master LightCycler^®^ 480 Probes kit (Roche Diagnostics, France) according to the manufacturer’s recommendations (Hoang et al., 2019). We amplified DNA of the following bacterial species: *Staphylococcus aureus, Streptococcus pneumoniae, Streptococcus pyogenes, Klebsiella pneumoniae, Moraxella catarrhalis, Enterococcus faecalis*, and *Haemophilus influenzae* based on their respective genes: *LPSau, lytA, hpt, phoE, CopB, recN*, and *OMPp1*, as previously described (Hoang et al., 2019). We developed a PCR in this study for the specific detection of *C. propinquum* targeting the DNA polymerase III subunit epsilon, and the primers used were Cprop_Dna3_MBF

TCACACTCACTGGCGAGTTC, Cprop_Dna3_MBR GACT ACCAGCACGGTGGTTT,

and Cprop_Dna3_MBP 6FAM- CCGGTTGTGGTCCGATA TCGC (Supplementary Table 1).

#### Routine Microbiological Analysis

Microbiological investigations included standard blood cultures (Dubourg et al., 2018), standard cultures of respiratory specimens (i.e., sputa, pleural fluids, bronchoalveolar lavages, bronchial aspirates, and pulmonary biopsies) (Dubourg et al., 2015), pneumococcal antigenic detection from urine specimens (Edelstein et al., 2020), and *S. aureus* PCR from bronchoalveolar lavage (BAL) (Oh et al., 2013). All positive results detected within 30 days following SARS-CoV-2 diagnosis were considered.

### Statistical Analysis

Baseline characteristics between COVID-19 positive and negative groups were compared using the Fisher Exact test (for categorical variables) and the Kruskal–Wallis test (for continuous variables). Data were analyzed using SPSS software (IBM, New York, United States). All *p*-values < 0.05 were considered significant. MicrobiomeAnalyst software (Chong et al., 2020) was used to analyze 16S ribosomal ribonucleic acid (rRNA) sequencing data, as previously described (Amrane et al., 2019). Linear discriminant analysis (LDA) of effect size (LEfSe) was performed using read counts (Edouard et al., 2018) and presence/absence to determine taxa differentially represented between COVID-19 positive (patients with a favorable outcome, asymptomatic, and deceased patients) and negative groups. Principal component analysis was used to visualize taxa profile and bacterial diversity between different groups (Amrane et al., 2019).

## Results

### Patients

A total of 120 pseudonymized samples were enrolled in this study, including COVID-19 negative subjects and 90 COVID-19 positive patients (Table 1). According to their clinical outcome, the COVID-19 group was divided into three subgroups of 30 patients each, including patients with a favorable outcome, asymptomatic, and deceased patients. Our study population was predominantly female, with a sex ratio (F/M) of 1.1 in favor of females. The oldest population were the deceased patients, with an overall mean age of 59.03 years.

**TABLE 1:** Clinical characteristics of patients with COVID-19 (*n* = 90) and COVID-19 negative group.

	COVID-19 patients			
Characteristics	With respiratory symptoms		Asymptomatic	COVID-19 negative group
	Favorable	Death		
Number of subjects	30	30	30	30
**Age (years)**				
Mean (*SD*)	46.4(15.4)	79.9(10.7)	46.7(18.7)	41.2(20.0)
Median [min–max]	44.5[22–77]	81[58–101]	44[10–79]	39[2–85]
Male (*n* = 55)	12	12	17	14
Female (*n* = 59)	12	18	13	16
Homeless (*n* = 6)	6	0	0	0

### General Composition of the Nasopharyngeal Microbiota

Among the 120 pseudonymized patients, we identified overall 1136 distinct OTUs (operational taxonomic unit) by 16S rRNA sequencing. Of these 1136 OTU, 214 were common to all groups, while 69 and 339 OTUs were only found in COVID-19 negative and COVID-19 positive subjects, respectively (Supplementary Figure 1). *Proteobacteria, Firmicutes*, and *Actinobacteria* were the phyla most identified, representing 64, 22, and 12%, respectively, of the reads generated among all groups. In asymptomatic COVID-19 patients, we observed an increase of *Proteobacteria* when compared to COVID-19 negative subjects (*p* ≤ 0.006), and a decrease of *Firmicutes* when compared to both deceased COVID-19 patients (*p* ≤ 0.04) and COVID-19 patients with a favorable outcome (*p* ≤ 0.004) (Supplementary Figure 2).

**FIGURE 1:**
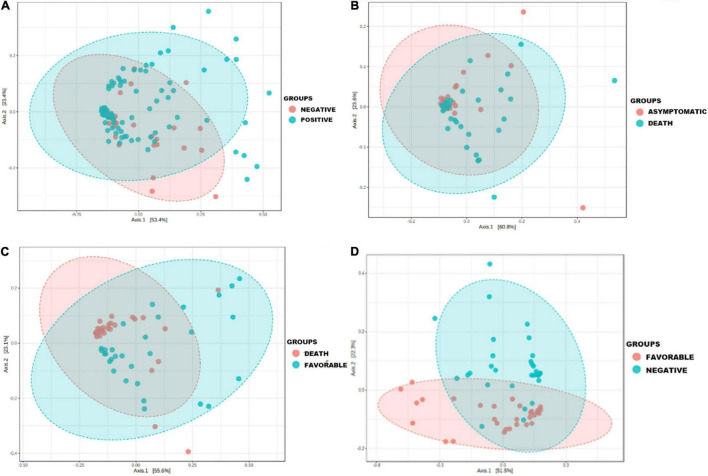
Principal coordinate analysis showing the change in the nasopharyngeal microbiota profile during COVID-19 airway infection in clinical patient groups versus COVID-19 negative group. Principal component analysis (PCoA) was performed on species from raw data (number of reads) using the Jensen Shannon divergence distance matrix and the Multivariate Analysis of Variance (MANOVA) statistical test. Each sample is represented by a point: **(A)** the microbiota profile of COVID-19 negative patients and cases (*p* ≤ 0.006); **(B)** the microbiota profile in asymptomatic patients and deceased patients (*p* ≤ 0.001); **(C)** the microbiota profile in patients with a favorable outcome and deceased patients (*p* ≤ 0.001); **(D)** the microbiota profile in patients with a favorable outcome and COVID-19 negative individuals (*p* ≤ 0.001).

The principal component analysis performed on the raw data generated in this study allowed us to observe that the nasopharyngeal microbiota composition was different according to the group studied (Figure 1).

The microbiota composition of most samples belonging to the positive group was far different than that belonging to the COVID-19 negative group (*p* ≤ 0.006) (Figure 1A), with a clear difference in the microbiota profile of deceased patients compared with patients in the favorable outcome group (*p* ≤ 0.001) (Figure 1C) and an approximation of the microbiota composition of some samples belonging to the favorable outcome group with that of other members of the negative group (*p* ≤ 0.001) (Figure 1D).

The number of reads per sample was significantly lower in COVID-19 negative subjects compared to COVID-19 patients (*p* ≤ 0.011), whether compared to that of the COVID-19 patients with a favorable outcome (*p* ≤ 0.0032) or deceased COVID-19 patients (*p* ≤ 0.063) subgroups (Supplementary Figure 4).

### Specific Airway Microbiota of COVID-19 Patients When Compared to That of Healthy Individuals

**FIGURE 2:**
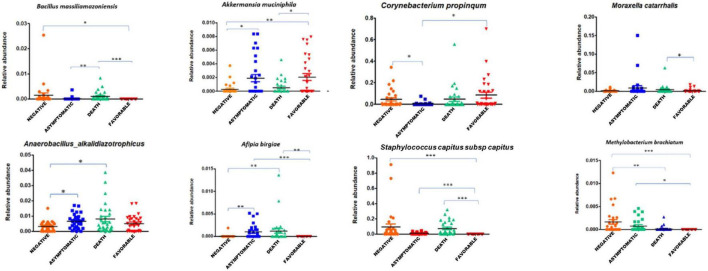
Association of specific taxa in the airway microbiota of COVID-19 patients. The relative abundance of differentially enriched taxa in the four clinical groups of COVID-19 patients (negative, asymptomatic, deceased patients, and patients with a favorable outcome). Five pathogens enrichment in groups of SARS-CoV-2 positive subjects: *Corynebacterium propinquum/pseudodiphtericum* (*p* ≤ 0.05), *Moraxella catarrhalis* (*p* ≤ 0.05), *Bacillus massiliamazoniensis* (*p* ≤ 0.01), *Anaerobacillus alkalidiazotrophicus* (*p* ≤ 0.05), and *Staphylococcus capitis* subsp. *capitis* (*p* ≤ 0.001). One-way ANOVA (Kruskal–Wallis statistic). *p ≤ 0.05, ^**^p ≤ 0.01, and ^***^p ≤ 0.001.

First, we found a significant decrease of anaerobic bacteria among COVID-19 positive patients when compared to the COVID-19 negative group (*p* < 0.029). Indeed, 25% (72/283) of the bacterial species enriched in the latter group were anaerobic, while 20% (66/323) were anaerobic in the asymptomatic subgroup, 19% (60/316) in the deceased group, and 13% (28/219) in patients in the favorable outcome subgroup. Using linear discriminant analysis (LDA), we observed six discriminating OTUs among COVID-19 positive patients, including *C. propinquum/pseudodiphtericum* (*p* ≤ 0.05), *M. catarrhalis* (*p* ≤ 0.05), *B. massiliamazoniensis* (*p* ≤ 0.01), *A. alkalidiazotrophicus* (*p* ≤ 0.05), *S. capitis* subsp. *capitis* (*p* ≤ 0.001), and *A. birgiae* (*p* ≤ 0.001) (Figure 2 and Supplementary Figure 3A).

In the COVID-19 negative group, *Methylobacterium brachiatum* (p ≤ 0.001), *Peptoniphilus gorbachi* (*p* ≤ 0.01), *Finegoldia magna* (*p* ≤ 0.05), and *Gemella haemolysans/sanguinis* (*p* ≤ 0.05) were increased when compared to cases (patients with a favorable outcome, asymptomatic, and deceased patients) (Supplementary Figure 3A).

### Correlation Between Indicator Taxa and the Clinical Course of the Disease

**FIGURE 3:**
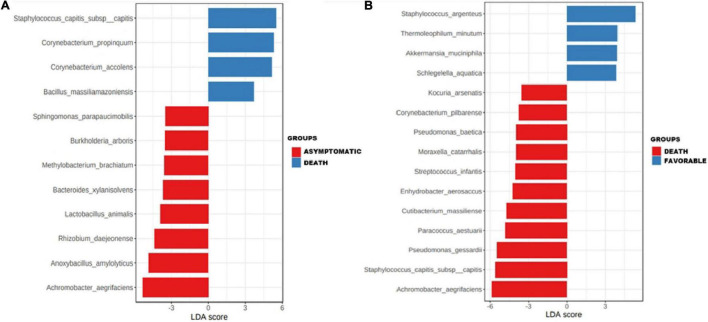
Association of specific taxa in the airway microbiota of COVID-19 patients. Enrichments of specific taxa in the respiratory microbiota of COVID-19 patients in asymptomatic and deceased patients **(A)** and in deceased patients and patients with a favorable outcome **(B)** by linear discriminant analysis (LDA) effect size (LEfSe).

Subgroup analysis of taxa showed that the species *S. capitis* subsp. *capitis* (*p* ≤ 0.001), *C. propinquum* (*p* ≤ 0.01), *C. accolens* (*p* ≤ 0.01), and *B. massiliamazoniensis* (*p* ≤ 0.05) were enriched among deceased patients when compared to the asymptomatic subgroup (Figure 3A). *Staphylococcus argenteus* (*p* ≤ 0.001), *Thermoleophilum minutum* (*p* ≤ 0.01), *Akkermansia muciniphila* (*p* ≤ 0.01), *Schlegelella aquatica* (*p* ≤ 0.01) were enriched in patients with a favorable outcome compared to deceased patients (Figure 3B).

### Pathogenic Bacteria Amplified by Real-Time PCR and Routine Clinical Microbiology

**FIGURE 4:**
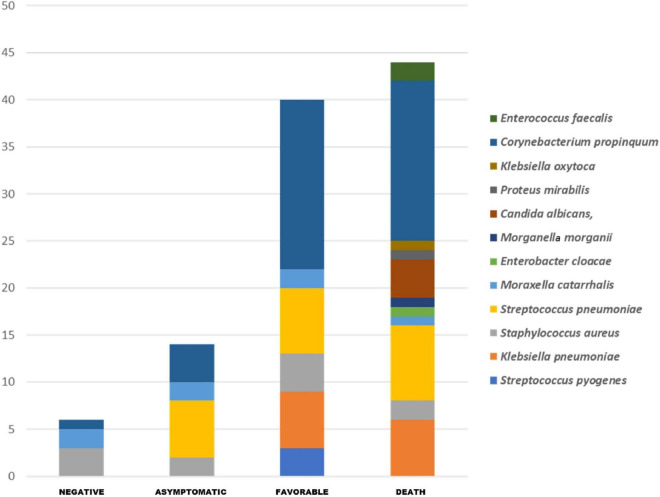
All respiratory pathogens routinely detected in our COVID-19 patients and by specific PCR. The pathogenic bacteria PCR shows the significant enrichment of *Streptococcus pneumoniae* (*p* ≤ 0.01) and *Corynebacterium propinquum* (*p* ≤ 0.000006) in the group of COVID-19 positive patients, and *Klebsiella pneumoniae* (*p* ≤ 0.01) in deceased patients and patients with a favorable outcome group compared to COVID-19 negative subjects, and *Enterococcus faecalis* (*p* ≤ 0.05) in deceased patients compared to negative individuals.

The PCR tests performed on nasopharyngeal swabs from each group revealed a significantly higher frequency of *S. pneumoniae* (*p* ≤ 0.01) in the COVID-19 group when compared to COVID-19 negative subjects, while *K. pneumoniae* were more frequent in deceased patients (*p* ≤ 0.01) and patients in the favorable outcome group (*p* ≤ 0.01) compared to negative individuals and *E. faecalis* (*p* ≤ 0.05) in deceased patients compared to the COVID-19 negative group (Figure 4). *C. propinquum* was more frequently detected among cases (97.5%) than among negative patients (2.5%) (*p* ≤ 0.000006). However, the detection rate of *C. propinquum* was lower among asymptomatic patients (10%) than among deceased patients (42.5%) or patients with a favorable outcome (45%) (Figure 4).

## Discussion

In the present study, we were able to show that SARS-CoV-2 infection is associated with a modification of the nasopharyngeal microbiota according to different stages of the disease, thus conferring on the microbiota a microbiological signature during the clinical evolution of the infection. First, we found a depletion of anaerobes among COVID-19 patients, irrespective of the clinical presentation of the infection. These results are in line with the study of Edouard et al. (2018) that included patients with respiratory infections involving various viruses, but not SARS-CoV-2. These data suggest that the loss of anaerobes could be a specific consequence of respiratory viral infections. We also highlighted an increase of Proteobacteria in asymptomatic COVID-19 patients in contrast to COVID-negative individuals and a considerable decrease of Firmicutes. These differences corroborate those observed by De Maio et al. (2020) who despite their analysis did not find statistically significant differences, which could be explained by their relatively smaller sample size (Table 2).

**TABLE 2:** The predominant nasopharyngeal microbiota in COVID-19 patients.

References	Patients	Methods	Results
Rosas-Salazar et al., 2021	*N* = 59 adults (38 with symptomatic, mild to moderate COVID-19+ and 21 asymptomatic, controls)	16S ribosomal RNA sequencing (V3–V4);	**Peptoniphilus lacrimalis, Campylobacter hominis, Prevotella 9 copri, and an *Anaerococcus* unclassified more abundant in SARS-CoV-2-infected and especially in those with high viral load. **Corynebacterium unclassified, *Staphylococcus haemolyticus*, *Prevotella disiens*, and 2 *Corynebacterium_1* unclassified more abundant in patients without SARS-CoV-2 or in those with low viral load.
De Maio et al., 2020	*N* = 40 (18 COVID-19+ symptomatic mild and 22 controls)	16S ribosomal RNA sequencing (V5–V6);	No major difference in the nasopharyngeal microbiota composition between COVID-19 patients and uninfected controls.
Zhang et al., 2020	*N* = 187 (62 patients with COVID-19 pneumonias and 125 with non-COVID-19 pneumonias)	RT-PCR and metatranscriptomic NGS Sequencing	The airway microbiome in COVID-19 patients with pneumonia had reduced alpha diversity, with 18 taxa of differential abundance when compared to patients with other pneumonias.
Mostafa et al., 2020	Nasopharyngeal (NP) swab specimens from *N* = 50 patients CoV 2019 disease (COVID-19)	Oxford Nanopore long-read third-generation metatranscriptomic and metagenomic sequencing.	Higher abundance of *Propionibacteriaceae* (*P* = 0.028) and a reduction in the abundance of *Corynebacterium accolens* (*P* = 0.025) among COVID-119 patients.
Lloréns-Rico et al., 2021	*N* = 58 COVID-19 patients (upper respiratory tract) *N* = 35 COVID-19 patients (lower respiratory tract)	16S ribosomal RNA sequencing (V4 region), Viral load determination and immunoprofiling	The microbiome (upper respiratory tract) of the entire cohort of COVID-19 patients was dominated by the Gram-positive genera Staphylococcus and Corynebacterium
This Study	Nasopharyngeal swab, *N* = 120 (90 with symptomatic, mild moderate and severe COVID-19+ and 30 healthy controls)	16S ribosomal RNA sequencing (V3–V4);	**Corynebacterium propinquum/pseudodiphtericum (*p* ≤ 0.05), *Moraxella catarrhalis* (*p* ≤ 0.05), *Bacillus massiliamazoniensis* (*p* ≤ 0.01), *Anaerobacillus alkalidiazotrophicus* (*p* ≤ 0.05), *Staphylococcus capitis* subsp. *capitis* (*p* ≤ 0.001) and *Afipia birgiae* (*p* ≤ 0.001) found enriched in COVID-19 patients when compared to COVID-19 negative subjects. **Staphylococcus capitis subsp. *capitis* (*p* ≤ 0.001), *Corynebacterium propinquum* (*p* ≤ 0.01), *Corynebacterium accolens* (*p* ≤ 0.01), and *Bacillus massiliamazoniensis* (*p* ≤ *0.05*) found enriched in COVID-19 deceased patients. **Methylobacterium brachiatum (*p* ≤ 0.001), *Peptoniphilus gorbachi* (*p* ≤ 0.01), *Finegoldia magna* (*p* ≤ 0.05), *Gemella haemolysans/sanguinis* (*p* ≤ 0.05) found enriched in COVID-19 negative subjects

**Significantly different.

These results are in line with other studies (Rosas-Salazar et al., 2021) that have observed a modification of the nasopharyngeal microbiota between symptomatic COVID-19 patients and negative subjects (Table 2).

Regarding the clinical course of the disease, we found a significant difference in the microbial profile of each sample grouped by severity index in the COVID-19 case groups, when compared to COVID-19 negative group (*p* ≤ 0.006), using principal component analysis. This diversity of the microbial community in the COVID-19 patient groups according to the severity index was observed by Mostafa et al. (2020) in a study conducted on nasopharyngeal swabs from 2 distinct groups of patients (a COVID-19 positive group and a COVID-19 negative group) (Table 2). Similar findings have been reported regarding infections involving other respiratory viruses (Edouard et al., 2018).

At the species level, we found 8 taxa enriched in the nasopharyngeal microbiota of COVID-19 positive patients when compared to COVID-19 negative subjects including *C. propinquum/pseudodiphtericum*, *M. catarrhalis, S. pneumoniae, K. pneumoniae*, and *E. faecalis.* Interestingly, these pathogens were previously found to be enriched in patients with respiratory infections involving other viruses (DeMuri et al., 2018; Edouard et al., 2018), suggesting that their overrepresentation may not be specific to COVID-19. However, a recent study conducted on a cohort of COVID-19 patients showed that the upper respiratory tract microbiome of COVID-19 patients is dominated by the *Corynebacterium* and *Staphylococcus* genera (Lloréns-Rico et al., 2021)(Table 2). In addition, *C. propinquum* and *C. accolens* belong to the 4 taxa enriched in deceased patients when compared to COVID-19 negative individuals. Interestingly, the latter was found to be more abundant in non-COVID-19 subjects when compared to COVID-19 patients in another study (Mostafa et al., 2020) (Table 2). *C. propinquum* was, however, much less detected among asymptomatic subjects than in patients who died or had a favorable outcome, raising the question of its involvement in the onset of symptoms. The genus *Corynebacterium* is predominant in the nasopharynx (Maxime et al., 2018) and the species *C. propinquum* primarily colonizes the nasopharyngeal region and is frequently isolated from respiratory specimens in patients with infection with pulmonary involvement (Riegel et al., 1993; Motomura et al., 2004). *S. pneumoniae*, which was increased among COVID-19 subjects, is a common respiratory pathogen and an agent of superinfections during viral episodes. Nasopharyngeal colonization with *S. pneumoniae* was associated with an impaired antiviral immune response in COVID-19 patients (Mitsi et al., 2022), while we did not observe this in our subgroups. *E. faecalis*, more frequent among deceased patients than in COVID-19 negative subjects, is surprisingly a main agent of bacteremia among COVID-19 patients, particularly in ICU settings (Giacobbe et al., 2021; Porto et al., 2022). Among the potentially protective species, we highlighted a decrease of *A. muciniphila* in deceased subjects when compared to those with a favorable outcome. To our knowledge, this species has been very rarely detected in respiratory samples but is well known for its anti-inflammatory properties, among others (Cani and de Vos, 2017). Finally, we have no explanation for the increase in Proteobacteria in asymptomatic subjects, as the presence of this phylum in the respiratory tract is frequently associated with inflammation or disease (Dickson et al., 2016).

Our study is comprehensive because it includes a large population size according to the clinical course of the disease, a total of 120 patients. We also acknowledge some limitations, such as the absence of a test for other respiratory viruses in our samples, which could have influenced our results. In addition, we cannot exclude that the changes in the composition of the nasopharyngeal microbiota observed in the present study are linked with alterations in the flora of other respiratory sites, such as the oral cavity or the lung. Indeed, the respiratory tract harbors a homogeneous microbiota whose biomass decreases from the upper to the lower tract (Charlson et al., 2011). However, the study demonstrates in the setting of COVID-19 a difference between upper respiratory tract bacteria obtained from nasopharyngeal swab and lower respiratory tract bacteria obtained from bronchoalveolar lavage (BAL) samples (Lloréns-Rico et al., 2021). Finally, we acknowledge that the analysis of the nasopharyngeal microbiota in our patients was performed after the diagnosis of COVID-19 disease. Therefore, we do not know if these changes were pre-existing before the SARS-CoV-2 infection. This is a topic for investigation, as the microbiota predisposing to respiratory viral infections is known from the literature for other pathogens (Allen et al., 2014; Maxime et al., 2018; Dubourg et al., 2019).

In summary, we have shown that the microbiota of COVID-19 patients may have a signature at different stages of the disease. Although we did not consider other respiratory infections during our evaluation, further studies considering co-infections with other viruses should be conducted to confirm whether several species, especially *C. propinquum*, could influence the course of the disease in order to include it as a predictor of COVID-19 severity. Nevertheless, we cannot state with certainty whether these bacteria will predict the development of secondary infections.

## Conclusion and Perspectives

**FIGURE 5:**
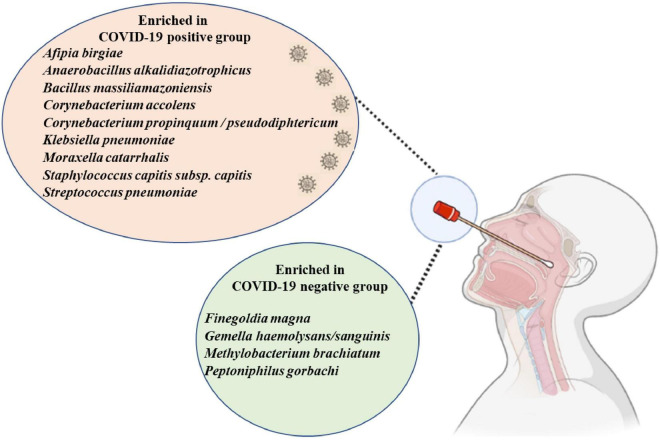
The enriched nasopharyngeal microbiota bacteria in the COVID-19 positive group and COVID-19 negative group. Generated using Biorender (https://biorender.com).

In this study, we were able to observe a specific microbial signature of the nasopharyngeal microbiota of COVID-19 patients (Figure 5), in particular a significant decrease in anaerobes. Among the species of interest, the role of *C. propinquum/diphteriticum, M. catarrhalis*, and *C. accolens* remains to be elucidated. As all COVID-19 patients were included during the first pandemic and were thereby infected with the same variant, it would be interesting to further investigate a possible correlation between the modification of the nasopharyngeal microbiota according to the different COVID-19 variants.

## Data Availability Statement

The datasets presented in this study can be found in the NCBI Sequence Read Archive under BioProject ID: PRJNA801106.

## Ethics Statement

This study consisted in studying pseudonymized samples not specifically obtained for this context but rather clinical samples remaining from diagnostic screenings. The patients were informed of the possible use of their samples for research purposes and retained their right to refuse approval at any point. According to the French Jardé Law (Law No. 2012–300 of March 05, 2012 and Decree No. 2016–1537 of November 16, 2016 published in the “Journal Officiel de la République Française”), as this study did not involve a specific collection of samples, neither institutional ethics approval nor individual patient consent was required for this non-invasive study. However, this general approach of retrospective study was validated by the Ethics Committee of the Méditerranée Infection Institute, under agreement no. 2022-011. The analysis of collected data followed the MR-004 reference methodology registered under no. 2020-152 and 2020-151 in the AP-HM register.

## Author Contributions

DR, J-CL, and GD conceived and designed the experiments. OT and VB contributed materials and analysis tools. OT, GD, and AY analyzed the data. OT and GD wrote the manuscript. J-CL and DR reviewed the manuscript. All authors approved the final version of the manuscript.

## Conflict of Interest

DR is a scientific board member of Eurofins company and a founder of a microbial culture company (Culture Top) and was a consultant for Hitachi High-Technologies Corporation, Tokyo, Japan, from 2018 to 2020. The remaining authors declare that the research was conducted in the absence of any commercial or financial relationships that could be construed as a potential conflict of interest.

## Publisher’s Note

All claims expressed in this article are solely those of the authors and do not necessarily represent those of their affiliated organizations, or those of the publisher, the editors and the reviewers. Any product that may be evaluated in this article, or claim that may be made by its manufacturer, is not guaranteed or endorsed by the publisher.

## Supplementary Material

Supplementary Figure 1 Venn diagram illustrating the different taxa specific to each group of COVID-19 patients.

Supplementary Figure 2 Relative abundance of phyla observed in all COVID-19 patients and in each group: an increase of *Proteobacteria* was observed in COVID-19 asymptomatic patients, compared to COVID-19 negative subjects (*p* ≤ 0.006). A decrease of *Firmicutes* was observed in COVID-19 asymptomatic patients compared to deceased patients (*p* ≤ 0.04) and patients with a favorable outcome (*p* ≤ 0.004).

Supplementary Figure 3 Association of specific taxa in the airway microbiota of COVID-19 patients. Enrichments of specific taxa in the respiratory microbiota of COVID-19 patients in **(A)** negative and COVID-19 patients (asymptomatic patients, deceased patients, and patients with a favorable outcome groups) and in **(B)** asymptomatic and deceased patients by presence-absence.

Supplementary Figure 4 The number of reads per sample representing each group of COVID-19 patients.

Supplementary Figure 5 All respiratory pathogens detected in our COVID-19 patients. Red: Specific real-time PCR. Green: Routine microbiological analysis.

Supplementary Table 1 Primers design: *Corynebacterium propinquum*.

## Abbreviations

ACE2: angiotensin converting enzyme 2
COVID: corona virus disease
DNA: desoxyribonucleic acid
IHU: Institut Hospitalo-Universitaire
LDA: linear discriminant analysis
LEfSe: linear discriminant analysis effect size
OTUs: operational taxonomic units
PCoA: principal coordinates analysis
PCR: polymerase chain reaction
rRNA: ribosomal ribonucleic acid
SARS-CoV-2: severe acute respiratory syndrome coronavirus 2.
