# Supplementary figures and images for: Profile of the Nasopharyngeal Microbiota Affecting the Clinical Course in COVID-19 Patients
Source: Front Microbiol. 2022 May 17;13:871627. doi: 10.3389/fmicb.2022.871627 (PMC9152678; doi:10.3389/fmicb.2022.871627)

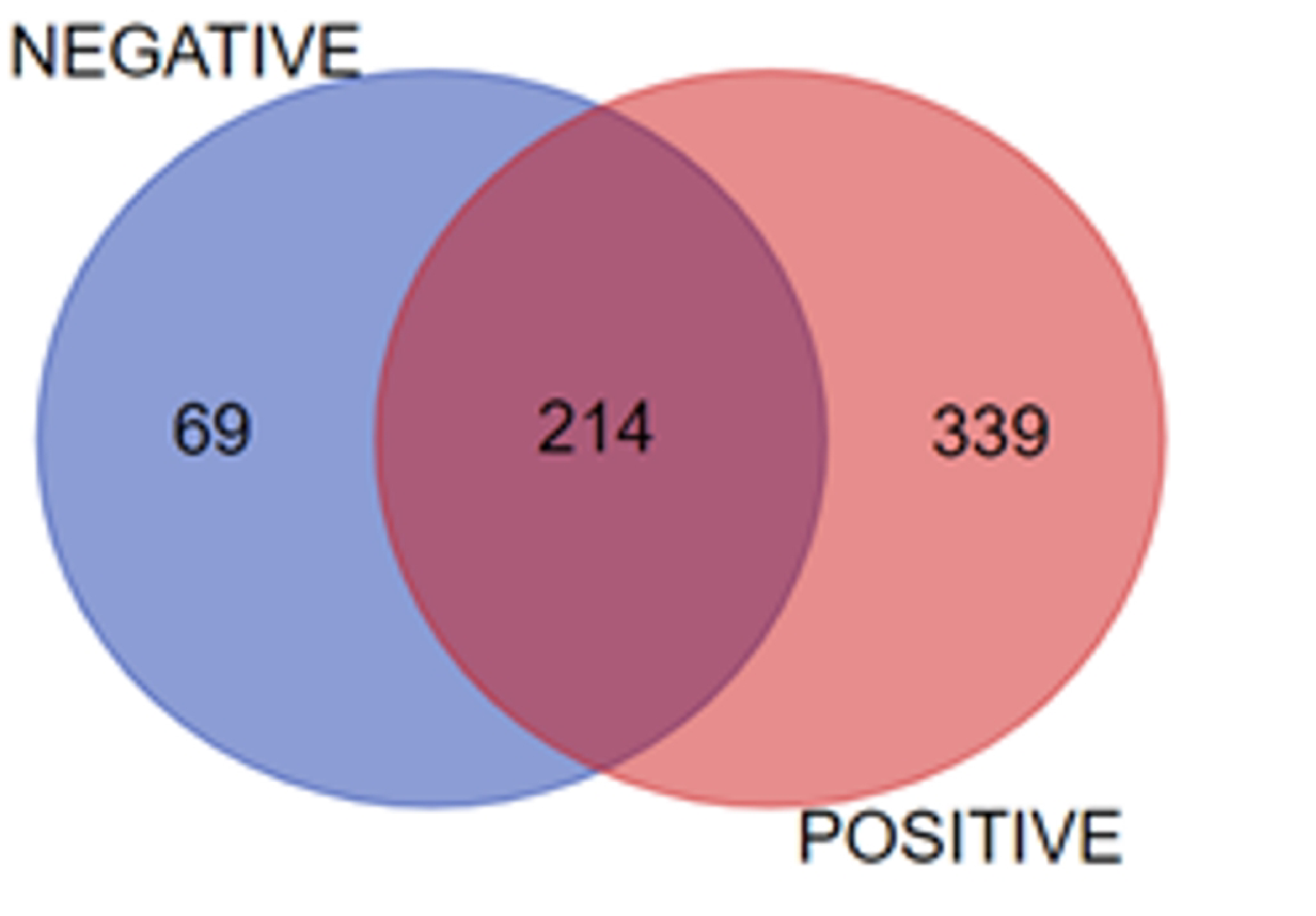

Supplement: Supplementary Figure 1 — Venn diagram illustrating the different taxa specific to each group of COVID-19 patients. [file Image_1.TIFF]

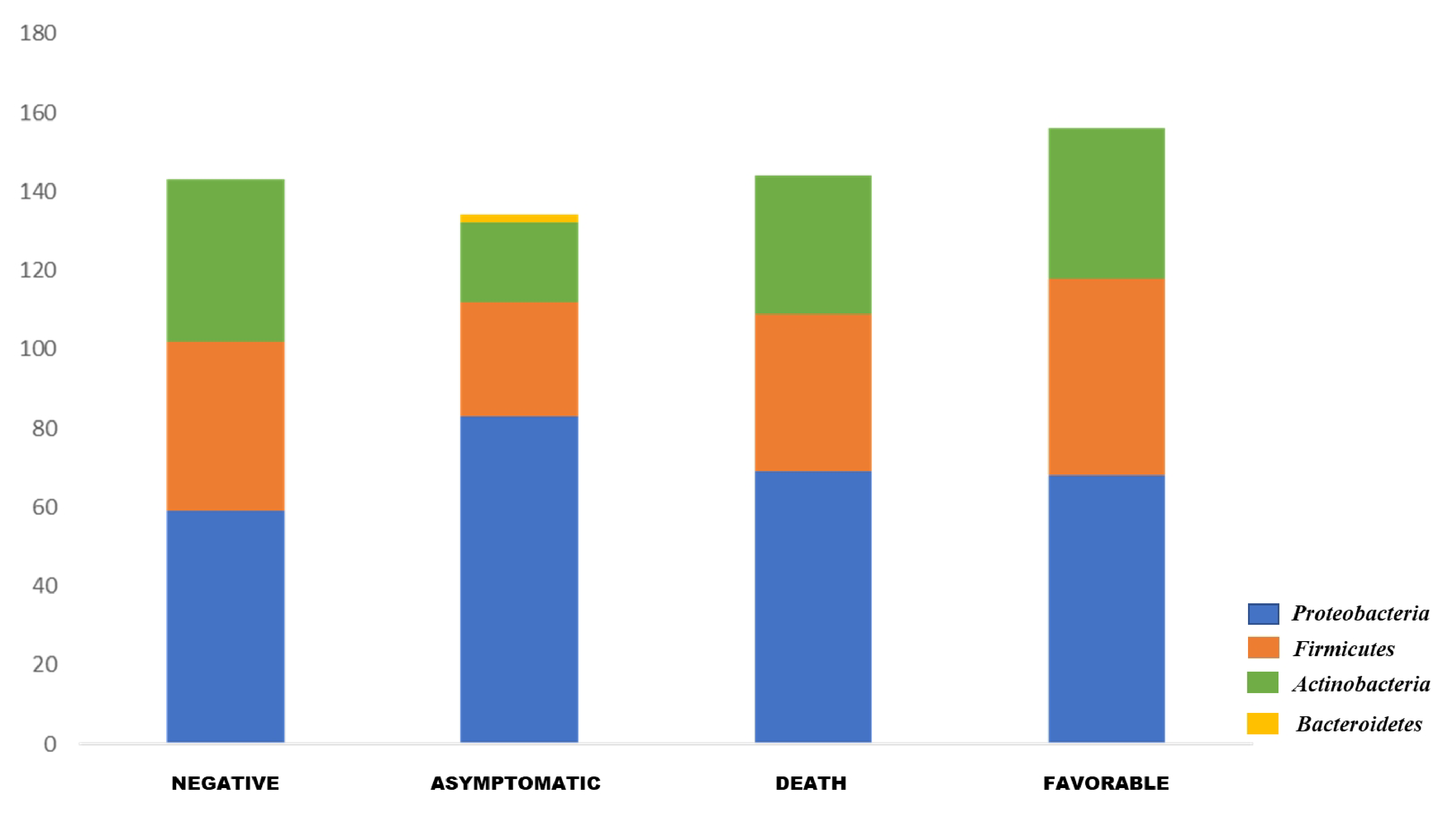

Supplement: Supplementary Figure 2 — Relative abundance of phyla observed in all COVID-19 patients and in each group: an increase of Proteobacteria was observed in COVID-19 asymptomatic patients, compared to COVID-19 negative subjects (p ≤ 0.006). A decrease of Firmicutes was observed in COVID-19 asymptomatic patients compared to deceased patients (p ≤ 0.04) and patients with a favorable outcome (p ≤ 0.004). [file Image_2.TIFF]

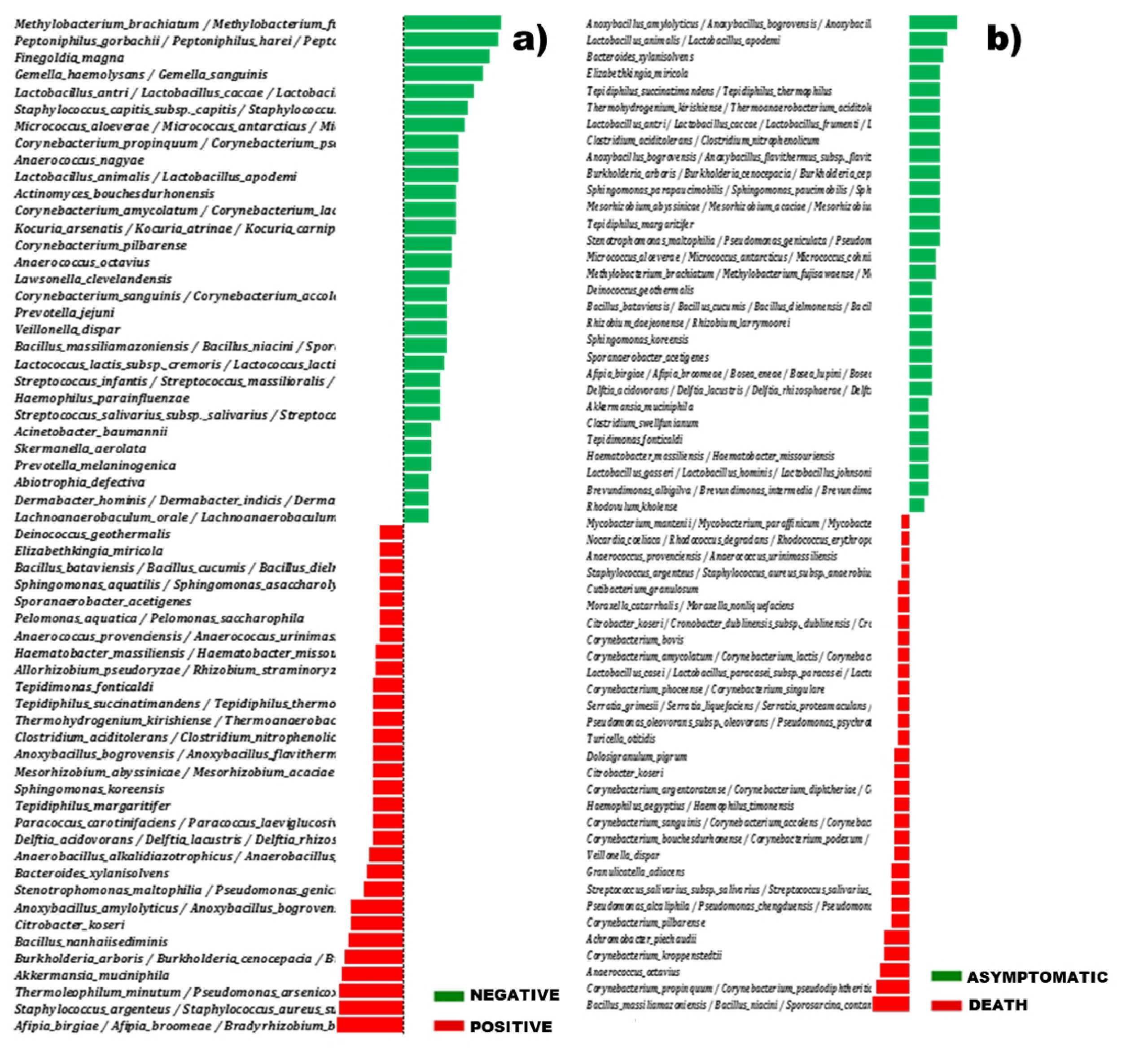

Supplement: Supplementary Figure 3 — Association of specific taxa in the airway microbiota of COVID-19 patients. Enrichments of specific taxa in the respiratory microbiota of COVID-19 patients in (A) negative and COVID-19 patients (asymptomatic patients, deceased patients, and patients with a favorable outcome groups) and in (B) asymptomatic and deceased patients by presence-absence. [file Image_3.TIFF]

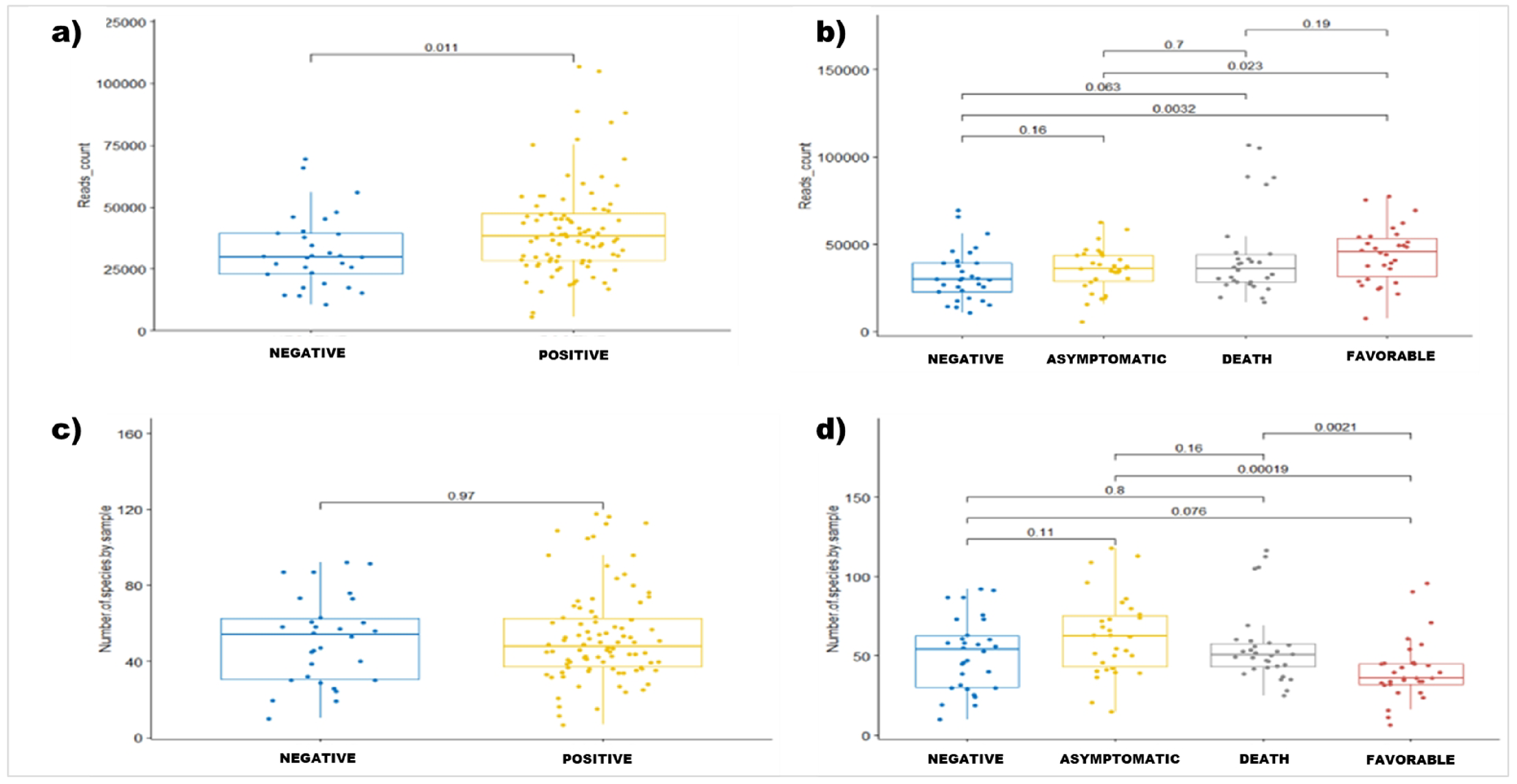

Supplement: Supplementary Figure 4 — The number of reads per sample representing each group of COVID-19 patients. [file Image_4.TIFF]

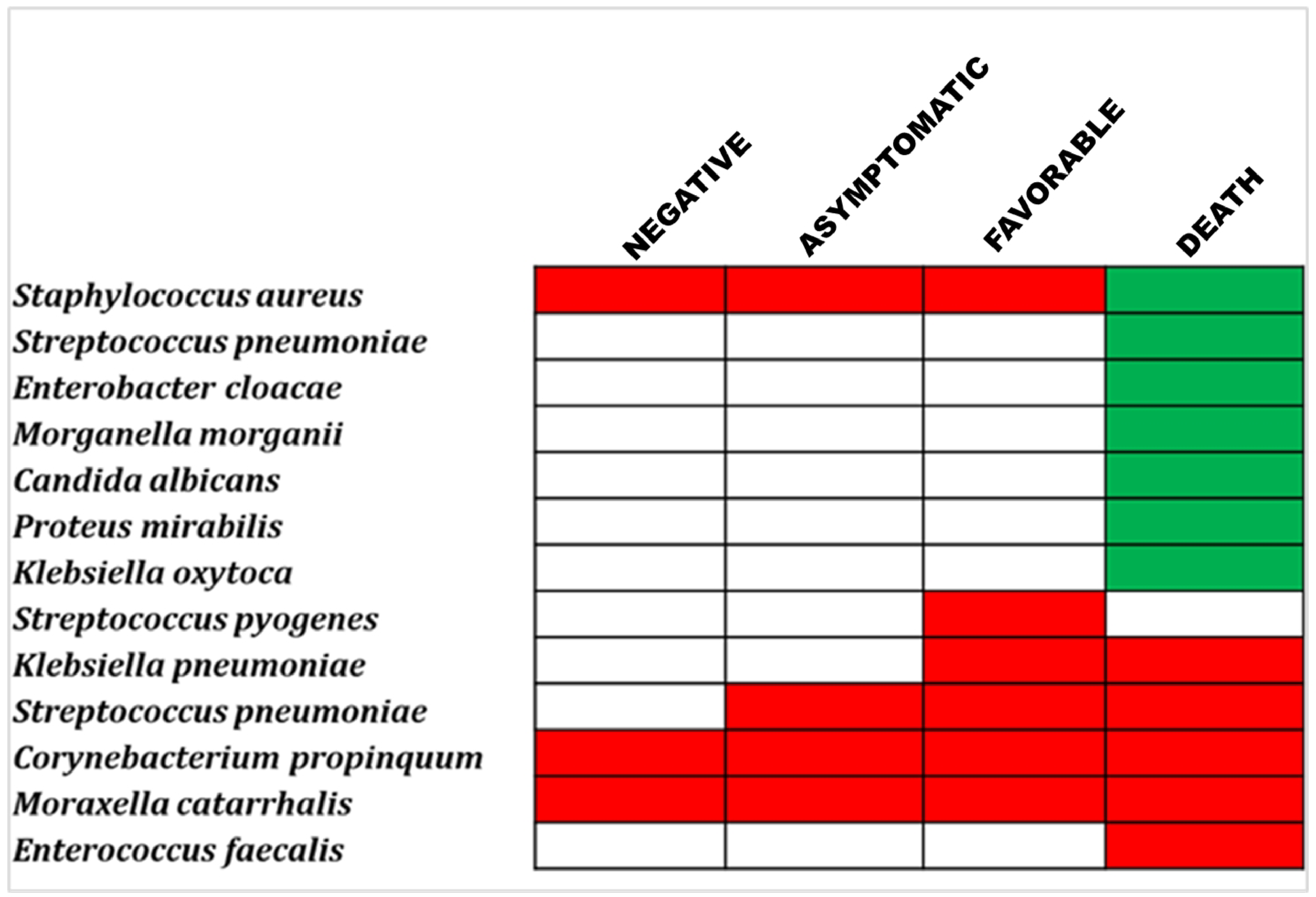

Supplement: Supplementary Figure 5 — All respiratory pathogens detected in our COVID-19 patients. Red: Specific real-time PCR. Green: Routine microbiological analysis. [file Image_5.TIFF]
