# Supplementary material for: Profile of the Nasopharyngeal Microbiota Affecting the Clinical Course in COVID-19 Patients
Source: Front Microbiol. 2022 May 17;13:871627. doi: 10.3389/fmicb.2022.871627 (PMC9152678; doi:10.3389/fmicb.2022.871627)
Supplement: Supplementary Table 1 — Primers design: Corynebacterium propinquum. [file Table_1.DOCX]

**Supp Table 1:** Primers design: *Corynebacterium propinquum*

| Targeted pathogen | Targeted sequence | Name primers | Number bases/primers | Sequences (5'-3') | °C sequence | Amplicon size (pb) |
| --- | --- | --- | --- | --- | --- | --- |
| *Corynebacterium propinquum* | DNA polymerase III subunit epsilon | Cprop_Dna3_MBF | 20 | TCACACTCACTGGCGAGTTC | 60 | 116 |
|  |  | Cprop_Dna3_MBR | 20 | GACTACCAGCACGGTGGTTT | 60 |  |
|  |  | Cprop_Dna3_MBP | 21 | 6FAM- CCGGTTGTGGTCCGATATCGC | 69 |  |
